# Percutaneous Transsplenic Balloon-Assisted Transjugular Intrahepatic Portosystemic Shunt Placement in Patients with Portal Vein Obliteration for Portal Vein Recanalization: Feasibility, Safety and Effectiveness

**DOI:** 10.1007/s00270-021-03054-2

**Published:** 2022-01-11

**Authors:** T. C. Meine, L. S. Becker, C. L. A. Dewald, S. K. Maschke, B. Maasoumy, E. Jaeckel, H. Wedemeyer, F. K. Wacker, B. C. Meyer, J. B. Hinrichs

**Affiliations:** 1 Institute for Diagnostic and Interventional Radiology, Hannover Medical School, OE8220 Carl-Neuberg-Straße 1, 30625 Hannover, Germany; 2 Department of Hepatology, Gastroenterology and Endocrinology, Hannover Medical School, Carl-Neuberg-Straße 1, 30625 Hannover, Germany

**Keywords:** Portal vein recanalization–Transjugular intrahepatic portosystemic shunt, Portal vein obliteration, Portal vein thrombosis, Transsplenic, Splenic access, Balloon puncture technique

## Abstract

**Purpose:**

To assess the feasibility, safety and effectiveness of portal vein recanalization (PVR)–transjugular portosystemic shunt (TIPS) placement via splenic access using a balloon puncture technique.

**Materials and Methods:**

In a single-center retrospective study from March 2017 to February 2021, 14 consecutive patients with portal hypertension, chronic liver disease and portal vein occlusion or near-complete (> 95%) occlusion were referred for PVR–TIPS placement. Feasibility, safety and effectiveness including procedural characteristics such as technical success, complication profile and splenic access time (SAT), balloon positioning time (BPT), conventional portal vein entry time (CPVET), overall procedure time (OPT), fluoroscopy time (FT), dose–area product (DAP) and air kerma (AK) were evaluated.

**Results:**

Transsplenic PVR–TIPS using balloon puncture technique was technically feasible in 12 of 14 patients (8 men, 49 ± 13 years). In two patients without detectable intrahepatic portal vein branches, TIPS placement was not feasible and both patients were referred for further treatment with nonselective beta blockers and endoscopic variceal ligation. No complications grade > 3 of the Cardiovascular and Interventional Radiological Society of Europe classification system occurred. The SAT was 25 ± 21 min, CPVET was 33 ± 26 min, the OPT was 158 ± 54 min, the FT was 42 ± 22 min, the DAP was 167.84 ± 129.23 Gy*cm^2^ and the AK was 1150.70 ± 910.73 mGy.

**Conclusions:**

Transsplenic PVR–TIPS using a balloon puncture technique is feasible and appears to be safe in our series of patients with obliteration of the portal vein. It expands the interventional options in patients with chronic PVT.

**Supplementary Information:**

The online version contains supplementary material available at 10.1007/s00270-021-03054-2.

## Introduction

Transjugular intrahepatic portosystemic shunt (TIPS) is an effective intervention to decompress portal hypertension and is recommended in patients with refractory ascites or gastrointestinal bleeding [1]. Portal hypertension due to portal vein thrombosis (PVT) might result in chronic obliteration of the portal vein (PVO), which causes morbidity and preclude patients from liver transplantation [2, 3]. TIPS creation is a complex procedure with high failure and complication rates in patients with PVO [2]. To overcome these challenges, recanalization of the portal vein (PVR) in combination with TIPS placement has been proposed [4]. Case series data suggest PVR–TIPS via splenic access has a high shunt patency, improves the survival of the patients and enables portal vein (PV) anastomosis in transplant candidates with chronic PVT [3–5]. However, there are a limited number of publications addressing technical aspects of the procedure [2, 4, 6]. We therefore sought to evaluate the feasibility, safety and efficacy of PVR–TIPS using a splenic access and a balloon puncture technique assessing the technical success, complication profile and peri-procedural characteristics.

## Materials and Methods

### Study Population

Between March 2017 and February 2021, 209 TIPS procedures were performed at our tertiary referral center and were retrospectively reviewed. Overall, 196 TIPS placements via a transjugular access were successful, which is also our standard approach in patients with PVT. In this period, 14 patients presented with chronic PVO defined as > 95% occlusion of the PV with or without portal cavernoma and PVT > 27 days after onset of symptoms [3, 7]. All patients underwent a standardized pre- and post-interventional workflow as described before and were included in the study [8].

### Transsplenic PVR–TIPS

**Fig. 1:**
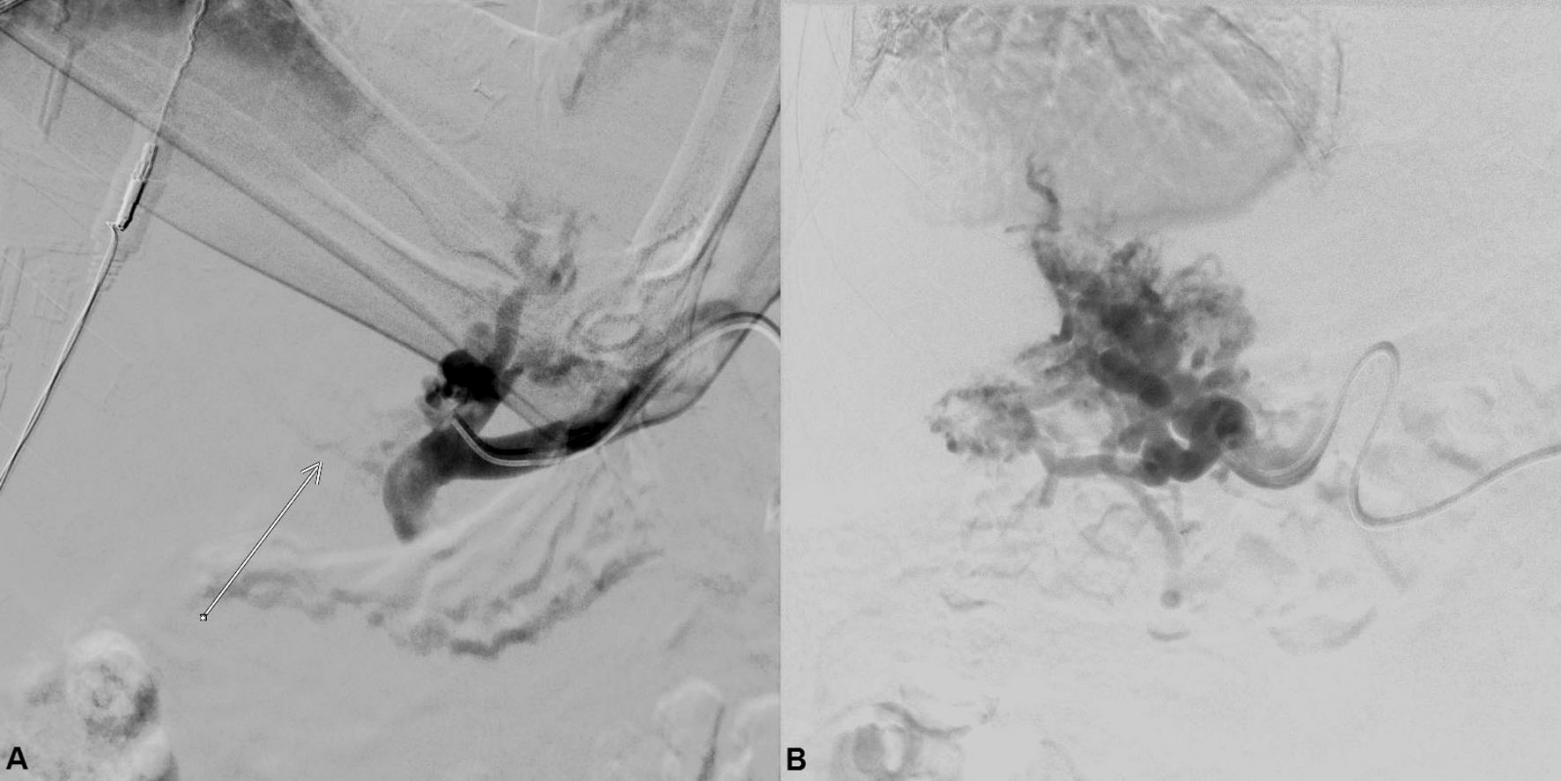
Splenoportogram of portal vein obliteration. **A** This splenoportogram was generated via a diagnostic catheter in the splenic vein. It shows the occlusion > 95% of the portal vein with a filiform residual portal vein branch (white arrow). Collateralization of the occlusion is conducted via esophagogastric varices. **B** In this digital subtraction angiography acquired via the splenic vein/portal confluence, a complete occlusion of the portal vein with cavernous transformation is present

**Fig. 2:**
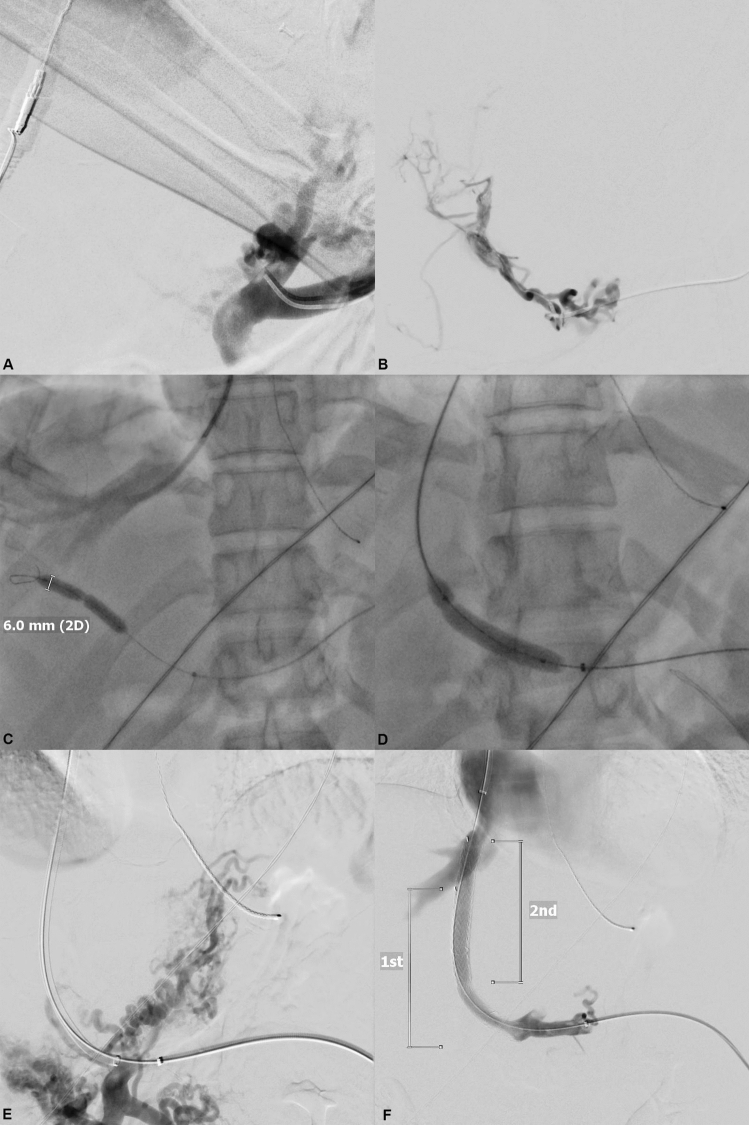
Balloon puncture technique for transsplenic portal vein recanalization–transjugular intrahepatic portosystemic shunt placement. **A** After successful splenic access was achieved, a 4-F diagnostic catheter was introduced in the splenic vein and a splenoportography the occlusion of > 95% of the portal vein was confirmed. **B** The obliterated portal vein was gently catheterized with a micro-catheter in this case and filiform original intrahepatic portal vein branches could be detected. **C** After recanalization of the portal vein, a 6-mm balloon catheter was inflated as the fluoroscopic target in the intrahepatic portal vein branch. The TIPS needle was introduced through a standard transjugular access into the appropriate hepatic vein. **D** After successful intrahepatic puncture under fluoroscopy guidance from the hepatic vein into the balloon-dilated portal vein branch, a control wire was introduced via the TIPS needle and is captured at the splenic access site. Then, a standard TIPS procedure was performed with gently dilatation of the puncture tract. **E** The TIPS sheath was advanced in through the TIPS tract and a portography was generated to evaluate the position of the TIPS stent graft. **F** A final portography was generated to confirm sufficient flow through the TIPS stent graft. In this case, a second TIPS stent graft was placed to cover the complete length of the TIPS tract

All transsplenic PVR–TIPS procedures were performed under general anesthesia by board-certified interventional radiologists (J.B.H., B.C.M.). First, a peripheral branch of the splenic vein (SV) was percutaneously punctured under ultrasound guidance with a micro-puncture set (4-F Custom Procedure Kit, Merit Medical). After successful SV access, a 4-F micro-puncture sheath was introduced. A 0.035-inch guidewire (150/260 cm, Terumo Corporation) was advanced and a 4-F sheath (Avanti® + , Cordis) was placed in the SV. Then, a 4-F diagnostic catheter (Berenstein, Impress, Merit Medical) was introduced in the SV and a splenoportogram was acquired with manual contrast injection to detect any residual PV branches (Fig. 1). The diagnostic catheter was gently advanced with the guidewire through the occluded portion of the PV or through a filiform portal cavernoma branch connected into an intrahepatic PV branch (Fig. 2). In this position, a balloon catheter (5/6 mmx40 mm, 135 cm, Sterling, Boston Scientific) was inflated as fluoroscopic target. Afterward, the TIPS needle (GORE TIPS Set, W.L.Gore&Associates) was advanced through a standard transjugular access into an appropriate hepatic vein and the balloon-dilated PV branch was punctured under fluoroscopy (Fig. 2). In case of successful puncture of the balloon cover, a 0.014-inch wire (V-14, Control Wire, Boston Scientific) was introduced through the TIPS needle and captured at the splenic access site. When the TIPS needle entered the PV branch without perforating the balloon cover, this was verified by aspiration of blood and injection of contrast media. Finally, a 0.035-inch guidewire was introduced through the transjugular access in the superior mesenteric vein or SV and a standard TIPS placement with a TIPS stent graft (GORE Viatorr TIPS Endoprosthesis with controlled Expansion, W.L.Gore&Associates) was performed (Fig. 2) [8].

### Data Collection and Statistical Analysis

Patient demographics and procedural characteristics including the complication profile according the Cardiovascular and Interventional Radiological Society of Europe (CIRSE) classification system were obtained from medical reports [8, 9]. The overall procedural time (OPT), fluoroscopy time (FT) and the dose–area product (DAP) were determined as defined elsewhere [8]. Furthermore, the splenic access time (SAT) as the puncture time of the SV confirmed by contrast media or an established guidewire in the SV branch and the balloon positioning time (BPT) as following the time until inflation of the balloon catheter as fluoroscopic target were assessed from interventional imaging. In addition, conventional portal vein entry time (CPVET) was determined as the time from the catheterized hepatic vein to the first documented image with the wire in the balloon cover or dilated PV branch. Finally, air kerma (AK) was recorded to evaluate the radiation exposure. Values are shown as mean ± standard deviation.

## Results

Patients’ demographics are described in detail in Table 1, and the procedural characteristics are given in Table 2 and Supplements. The transsplenic PVR–TIPS via balloon puncture technique was technically successful in 12 of 14 patients. In two patients with portal cavernoma, no intrahepatic PV branch could be detected at the time of intervention and the procedure was aborted. Both patients were referred for further treatment with nonselective beta blockers and/or endoscopic variceal ligation and are under surveillance. No complication grade > 3 of the CIRSE classification system occurred. Grade 3 complications were treated with blood transfusion (one subcapsular splenic hematoma), antibiotic medication (one spontaneous bacterial peritonitis) and compression (two secondary access site bleedings).

**Table 1:** Patient’s characteristics

Number	14
Age (years)	49 ± 13
Sex (male/female)	8/6
MELD score	10 ± 6
Liver disease	
Alcohol-induced	5
HBV	1
AIH	2
PSC	1
SSC	1
FNH/Adenoma	2
Congenital fibrosis	1
Cryptogenic	1
Complications of portal hypertension	
Refractory variceal or gastrointestinal bleeding	10
Refractory ascites and hydrothorax	4
Portal cavernoma	8

Age, sex, model of end-stage liver disease (MELD) score, liver disease and complication of portal hypertension are tabulated for the study population. One patient with focal nodular hyperplasia had essential thrombocytosis as another cause for portal hypertension

*HBV* hepatitis virus B, *AIH* autoimmune hepatitis, *PSC* primary sclerosing cholangitis, *SSC* secondary sclerosing cholangitis, *FNH* focal nodular hyperplasia

**Table 2:** Procedural characteristics

Technical success	12/14
Additional procedures	
Second stent-graft placement	6
Variceal embolization	4
Complications according CIRSE classification system	
1	7/14
2	0/14
3	3/14
4	0/14
5	0/14
6	0/14
Procedural times	
Splenic access time (min)	25 ± 21
Balloon positioning time (min)	55 ± 35
Conventional portal vein entry time (min)	33 ± 26
Overall procedural time (min)	158 ± 54
Radiation exposure	
Fluoroscopy time (min)	42 ± 22
Dose–area product (Gy*cm2)	167.84 ± 129.23
Air kerma (mGy)	1150.70 ± 910.73

Procedural characteristics for the study populations included technical success rate, additional procedures, complications, procedural times and radiation exposure. In one patient, the balloon puncture time could not be determined due to lack of data

*CIRSE* Cardiovascular and Interventional Radiological Society of Europe

## Discussion

PVR–TIPS placement using a splenic access has been shown to improve survival and transplant candidacy in patients with chronic PVT [5], but it is still challenging due to the lack of PV target. Fluoroscopic targets within the obliterated PV branch have been reported to be useful [3, 4]. Habib et al. advanced a snare device through a splenic access in the PV branch to guide the intrahepatic PV puncture [3]. In contrast, Chen et al. used a balloon catheter as fluoroscopic target in the PV branch via a transhepatic approach [6]. We combined both approaches using a splenic access and a balloon catheter to assist PVR–TIPS placement, because the balloon catheter might serve as a larger fluoroscopic target in an obliterated PV branch as the snare device might not fully deploy.

Procedural characteristics for transsplenic PVR–TIPS are only available from Habib’s study [4]. The technical success rate in our study with 85% was slightly inferior to Habib et al. with 100% [3]. In two cases in our study, no intrahepatic PV branch could be identified at the time of the intervention. The final interventional option in these patients might be the creation of an extrahepatic or extracorporeal portosystemic shunt, e.g., via a transmesenteric or direct percutaneous access [10–12].

No major complications occurred in our study. The reported complications in our study required no further interventional or surgical treatment, which reflects the safety of our technique.

The effectiveness of the balloon puncture technique can be analyzed using the FT [13]. The mean FT of 42 min in our study is even lower than the reported FT of 53 min for the snare puncture technique although the group from Chicago did not perform variceal embolization [3]. The dilatation of the PV branch with the balloon as a fluoroscopic target offers visual and haptic feedback that might accelerate the puncture compared to the snare puncture technique.

Considering the procedural times, the mean OPT of 158 min of our transsplenic balloon-assisted PVR–TIPS procedure is comparable to the snare puncture technique with a mean procedural time of 147 min [3]. Time-consuming steps include splenic access and probing the obliterated PV branch. Catheterization of the SV and dilatation of the intrahepatic PV branch account for 30% of the OPT. Finally, the relatively high radiation exposure in our study is still below the TIPS reference level [14]. Focused on the procedural characteristics, the SAT was shorter than the BPT, but the CPVET was not increased in relation to the literature [8]. OPT, FT, DAP and AK were comparably high for TIPS procedures [8].

Small sample size and retrospective study design are study limitations. Furthermore, no follow-up data for this cohort were assessed pertaining clinical outcomes and patency rates.

## Conclusion

Overall, transsplenic PVR–TIPS using a balloon puncture technique was technically feasible and appears to be safe. It is an alternative to the reported snare puncture technique in patients with chronic PVT.

## Supplementary Information

Below is the link to the electronic supplementary material.

Supplementary file1 (DOCX 18 kb)

## Abbreviations

AK: Air Kerma
BPT: Balloon Positioning Time
CIRSE: Cardiovascular and Interventional Radiological Society of Europe
CPVET: Conventional Portal Vein Entry Time
DAP: Dose–Area Product
FT: Fluoroscopy Time
MELD: Model of End-Stage Liver Disease
OPT: Overall Procedural Time
PV: Portal Vein
PVO: Portal Vein Obliteration
PVR: Portal Vein Recanalization
PVT: Portal Vein Thrombosis
SAT: Splenic Access Time
SV: Splenic Vein
TIPS: Transjugular Intrahepatic Portosystemic Shunt
