# Supplementary material for: Percutaneous Transsplenic Balloon-Assisted Transjugular Intrahepatic Portosystemic Shunt Placement in Patients with Portal Vein Obliteration for Portal Vein Recanalization: Feasibility, Safety and Effectiveness
Source: Cardiovasc Intervent Radiol. 2022 Jan 11;45(5):696–702. doi: 10.1007/s00270-021-03054-2 (PMC9018628; doi:10.1007/s00270-021-03054-2)
Supplement: Supplementary file 1 — Supplementary file1 (DOCX 18 kb) [file 270_2021_3054_MOESM1_ESM.docx]

**Supplements**

|  |  | **Procedural Times** | | | **Radiation exposure** | | | **Additional Procedures** | | **Complication Profile** | |
| --- | --- | --- | --- | --- | --- | --- | --- | --- | --- | --- | --- |
| **Patient No.** | **SAT**  min | **BPT**  min | **CPVET**  min | **OPT**  min | **FT**  min | **DAP**  Gy*cm2 | **AK**  mGy | **Number of Stents** | **Variceal embolization** | **Complication** | **CIRSE classification system** |
| **1** | 33 | 77 | 32 | 203 | 52 | 198.29 | **1246.00** | 2 | 1 Plug | Dissection of a cardiofundal vein | 1 |
| **2** | 20 | 25 | 4 | 100 | 27 | 65.69 | **364.30** | 1 | 2 Plugs | Bile duct puncture | 1 |
| **3** | 11 | 28 | 78 | 151 | 60 | 215.87 | **1649.34** | 2 | 0 | - | - |
| **4** | 13 | 65 | 26 | 173 | 47 | 212.04 | **1457.34** | 1 | 0 | Bile duct puncture | 1 |
| **5** | 20 | 23 | 21 | 110 | 27 | 213.52 | **1799.34** | 1 | 0 | Hepatic artery puncture | 1 |
| **6** | 11 | 37 | 24 | 109 | 26 | 42.96 | **287.39** | 1 | 2 Plugs | - | - |
| **7** | 21 | 71 | 15 | 171 | 34 | 76.76 | **455.64** | 2 | 0 | Access site bleedings | 3 |
| **8** | 31 | 32 | 15 | 124 | 30 | 140.54 | **715.71** | 2 | 0 | Bile duct puncture | 1 |
| **9** | 20 | 119 | 84 | 268 | 92 | 345.89 | **2317.51** | 2 | 2 Plugs | Extracapsular puncture | 1 |
| **10** | 16 | 29 | 52 | 164 | 35 | 59.75 | **488.11** | 2 | 0 | Bile duct puncture | 1 |
| **11** | 16 | 40 | 3 | 98 | 13 | 6.58 | **55.97** | 1 | 0 | Spontaneous bacterial peritonitis | 3 |
| **12** | 90 | 123 | 37 | 230 | 61 | 436.12 | **2971.76** | 1 | 0 | Subcapsular spleen hematoma | 3 |

Procedural characteristics included procedural times, radiation exposure, additional procedures and complication profile and were tabulated for the every patient. Times were given in minutes, dose-area-product (DAP) in Gy*cm^2^ and air kerma (AK) in mGy. Abbreviations: SAT = splenic access time, BPT = balloon positioning time, CPVET = conventional portal vein entry time, OPT = overall procedural time, FT = fluoroscopy time and CIRSE = Cardiovascular and Interventional Radiological Society of Europe.
